# Delayed Diagnosis of Unilateral Absence of Pulmonary Artery Presenting with Recurrent Bronchopneumonia

**DOI:** 10.1016/j.case.2023.12.004

**Published:** 2024-03-08

**Authors:** Raja Ezman Raja Shariff, Mohd Rahal Yusoff, Khairul Shafiq Ibrahim, Sazzli Kasim

**Affiliations:** Cardiology Department, Universiti Teknologi Mara (UiTM) Sungai Buloh, Sungai Buloh, Selangor, Malaysia

**Keywords:** Unilateral absence of pulmonary artery, Adult congenital heart disease, Complications, Case report

## Abstract

• Unilateral absence of the pulmonary artery (UAPA) is a rare congenital condition.
• Patients with UAPA may present initially following recurrent bouts of pneumonia.
• Echocardiography remains a useful tool for diagnosis in resource-limited settings.

## Graphical abstract

**Figure:**
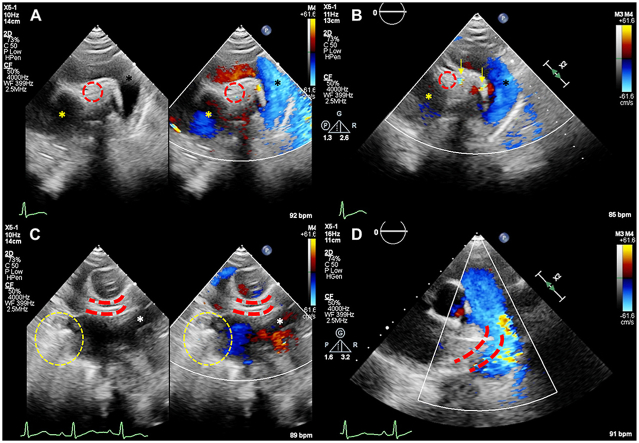

## Introduction

Unilateral absence of pulmonary artery (UAPA) is a rare congenital condition that can often be misdiagnosed due to varying clinical presentations. Although multimodality imaging is often pivotal in confirming the diagnosis, echocardiography provides a useful tool to identify supportive features of the diagnosis, especially in resource-limited settings. We report a young patient with newly diagnosed isolated UAPA and with recurrent admissions for bronchopneumonia over a decade.

## Case Presentation

A 31-year-old woman presented to our emergency department in a district hospital with worsening dyspnea and wheeze over a 48-hour period. The patient was diagnosed with possible bronchial asthma 15 years ago and experienced recurrent hospital admissions for assumed exacerbation of bronchial asthma over the past decade. The patient had chest radiographs performed from previous admissions that were unobtainable as they had been performed in other health care facilities outside our district.

Clinical examination revealed a respiratory rate of 30 breaths/minute, oxygen saturation of 88% on room air requiring oxygen supplementation, and temperature of 38°C. There were diffuse rhonchi on auscultation of the lungs, with reduced breath sounds and coarse crepitations over the left lower zone. The patient was initially treated with nebulized salbutamol in the emergency department with little improvement and subsequently required intubation and mechanical ventilation. The patient was also started on intravenous piperacillin-tazobactam for possible bronchopneumonia and asthma exacerbation.

**Figure 1:**
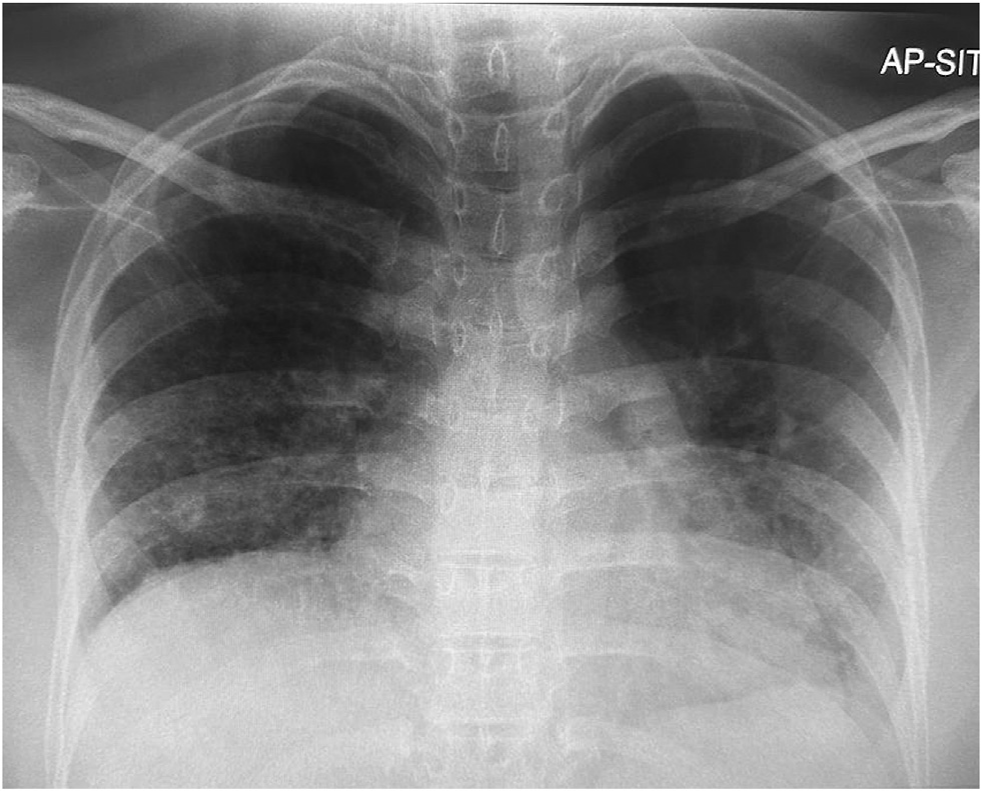
Chest radiogram, anterior-posterior projection, demonstrates diffuse reticular shadowing with reduced lung volume on the right side with a raised right-sided hemidiaphragm and compensatory hyperinflation and plethora on the left hemithorax.

Chest radiography demonstrated diffuse reticular shadowing (Figure 1). Blood and sputum cultures grew methicillin-resistant *Staphylococcus aureus*, prompting an abdominal ultrasound and transthoracic echocardiogram (TTE) to exclude systemic dissemination.

**Figure 2:**
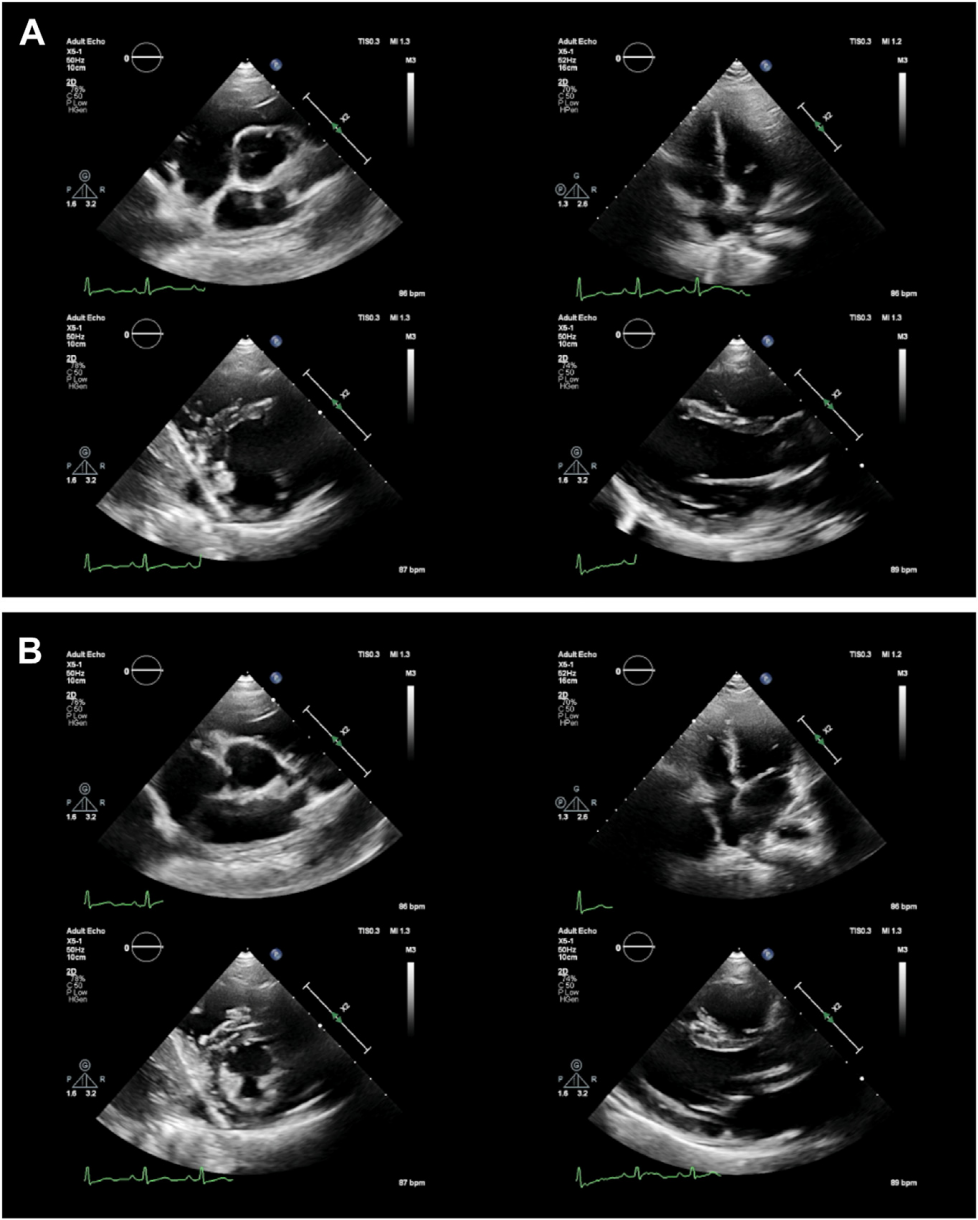
Two-dimensional TTE, basal parasternal short-axis (PSAX; *top left*), apical 4-chamber (*top right*), mid PSAX (*bottom left*), and parasternal long-axis (*bottom right*) views in **(A)** diastole and **(B)** systole reveal normal LV systolic function (LV ejection fraction 56%), normal wall thickness, and normal right heart dimensions.

**Figure 3:**
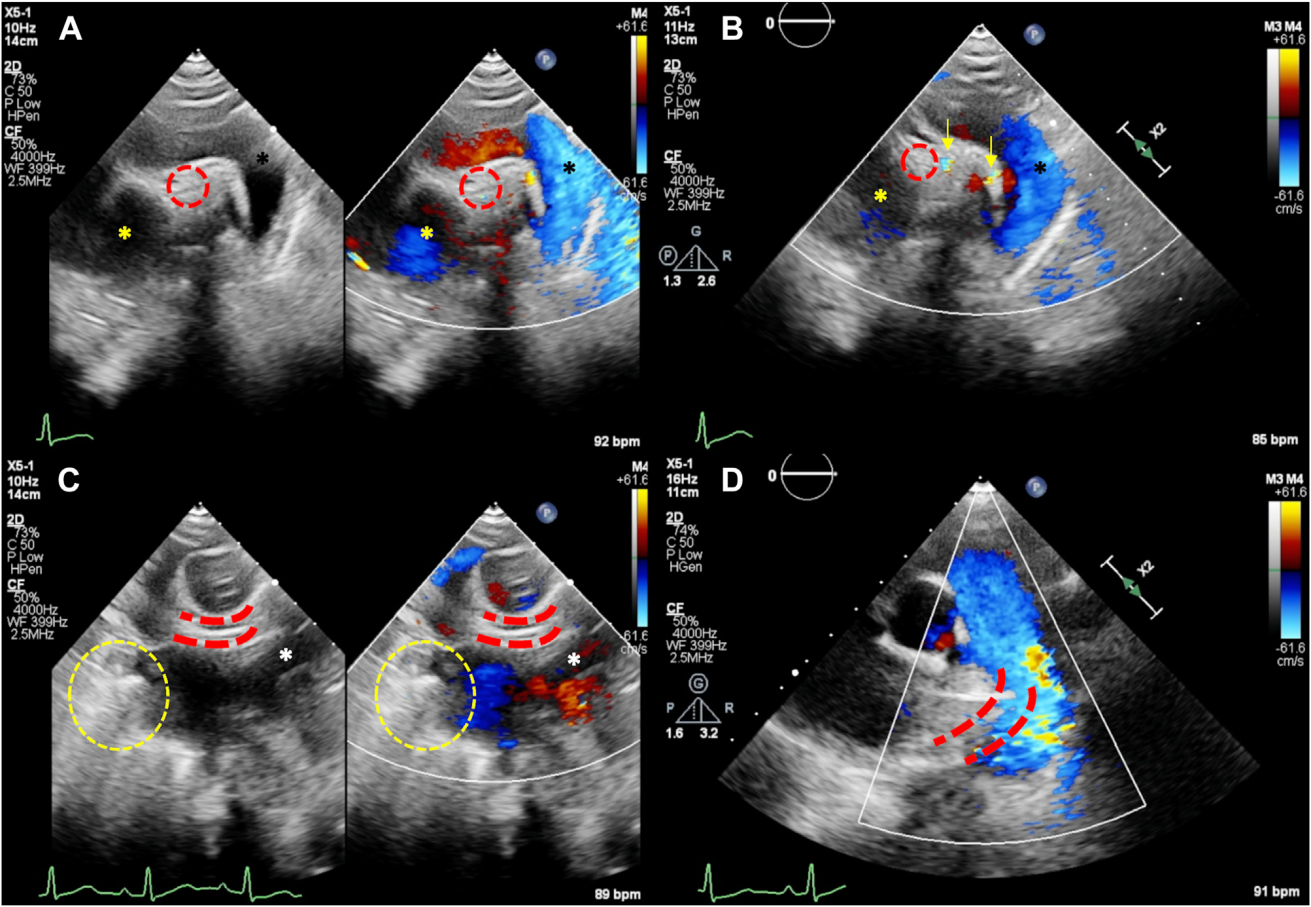
Two-dimensional TTE without (*left*) and with (*right*) color-flow Doppler, suprasternal view **(A)**, demonstrates the LA (*yellow asterisks*), descending aorta (*black asterisks*), and absence of the RPA, which should be located where the *red dotted circles* are placed. **(B)** There is evidence of flow within the left main bronchus (*yellow arrows*) suggestive of collateral blood flow (later confirmed to be bronchial artery collateral flow on CT imaging). **(C)** From the crab view window, the left pulmonary veins (*white asterisks*) were visible, but there was absence of the right pulmonary veins, which should be located where the *yellow dashed circles* are placed. The RPA was also absent, which should be located where the *red dotted lines* are placed. **(D)** On the high parasternal short-axis view, there was missing divergent flow from the main pulmonary artery to the RPA, which should be located where the *red dotted lines* are placed.

Transthoracic echocardiography revealed a normal left ventricular (LV) systolic function, with normal wall thickness and LV ejection fraction of 56% using the biplane Simpson’s method (Figure 2, Video 1, Video 2, Video 3, Video 4). There was also a normal right ventricle (RV) with basal, mid, and longitudinal diameters measuring 3.5, 2.9, and 6.5 cm, respectively, and proximal and distal RV outflow tract diameter measuring 2.8 and 2.4 cm, respectively, with a normal RV wall thickness of 0.3 cm. Both the left (LA) and right atria (RA) were of normal dimensions as well (LA volume index of 21 mL/m^2^ and RA volume index of 12 mL/m^2^). There was no overt valvular lesion seen during the TTE. There was, however, absence of the right pulmonary artery (RPA) noted on the parasternal short-axis view. Further views using a suprasternal window demonstrated a similar absence of the RPA alongside turbulent flow suggestive of collateral circulation (Figure 3). A crab view window was then performed (by rotating the transducer 90° clockwise and tilting both anteriorly and inferiorly), which revealed absent neighboring right pulmonary veins alongside UAPA (Figure 3, Videos 2, 5, and 6). There was no evidence on TTE to suggest possible pulmonary hypertension, with only mild tricuspid regurgitation (peak velocity of 2.0 m/s) and no evidence of pulmonary regurgitation.

**Figure 4:**
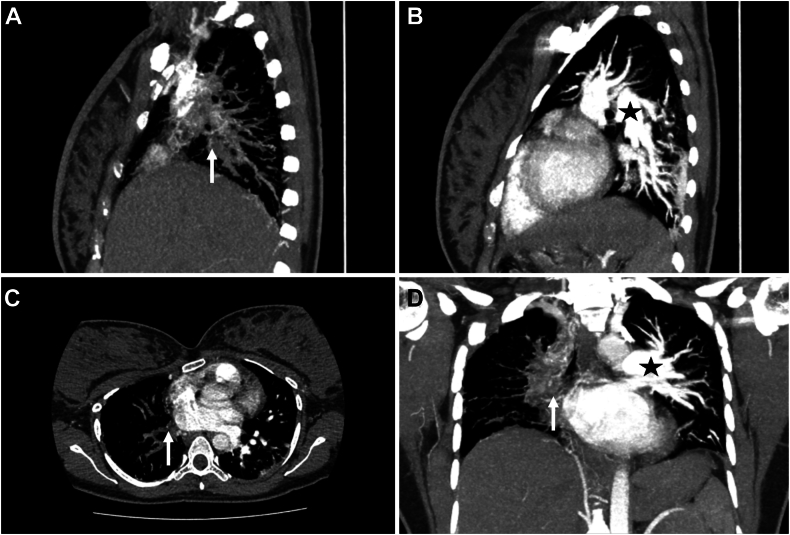
Chest CT with contrast demonstrates a filling defect seen where an RPA (*white arrows*) should arise from the main pulmonary artery trunk in the **(A)** right lateral sagittal, **(B)** left lateral sagittal, **(C)** axial, and **(D)** coronal planes. For comparison, the left pulmonary artery (*black stars*) can be seen.

**Figure 5:**
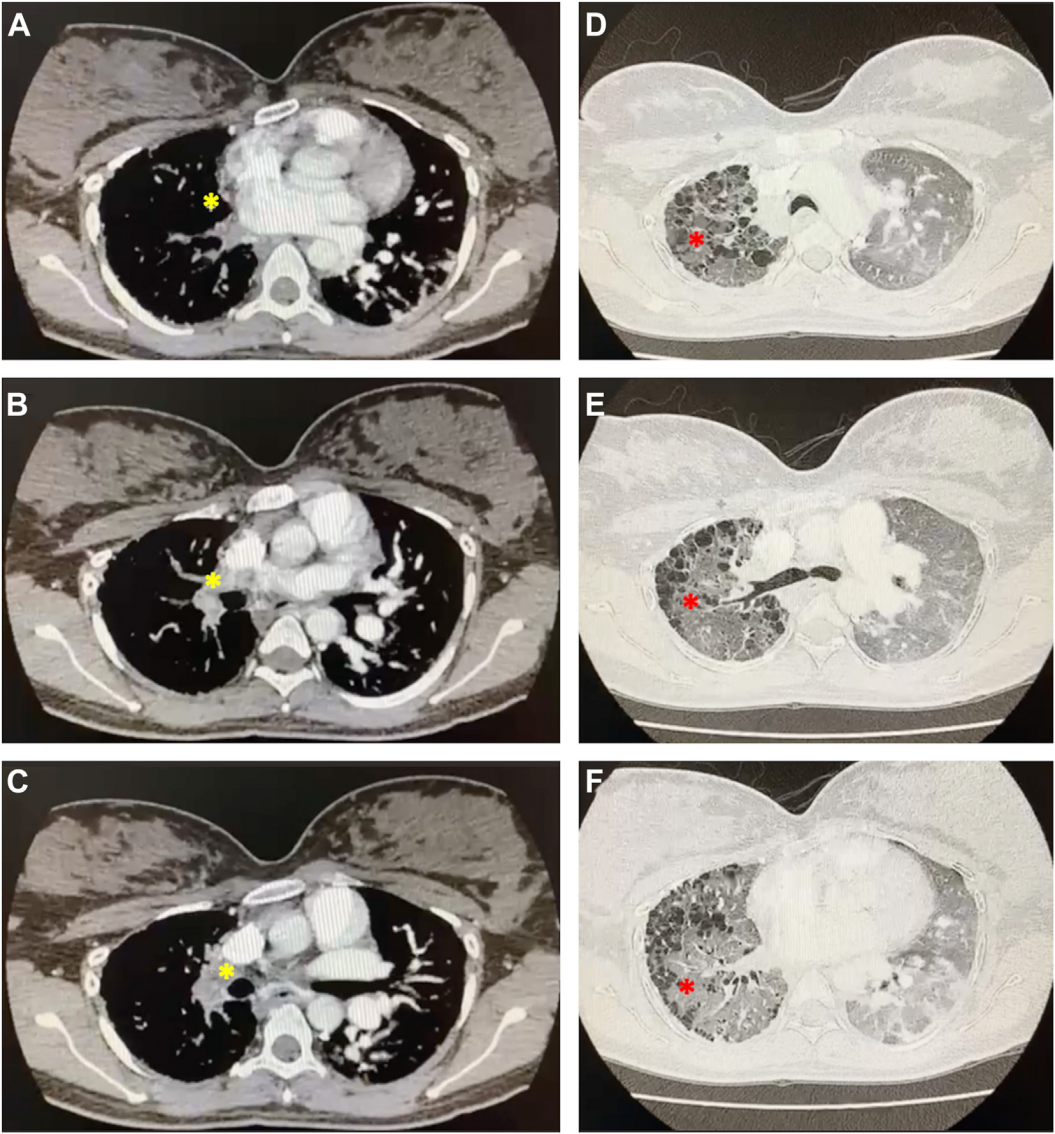
Chest CT with contrast, serial axial images from caudal to cranial **(A-C)**, demonstrates filling defects where an RPA should arise from the main pulmonary artery trunk (*yellow asterisks*). **(D-F)** Using the lung window, there is evidence of a hypoplastic right lung with multiple clustered lung cysts of peripheral and peribronchovascular distribution (*red asterisks*), with left lung compensatory hyperinflation, pulmonary plethora, and focal consolidation of the posterior segment of the left lower lobe suggestive of acute infective changes.

Computed tomography (CT) imaging of the thorax was subsequently performed confirming the unilateral absence of the RPA with multiple ipsilateral collaterals from the main pulmonary trunk and bronchial arteries (Figure 4). There was also evidence of a hypoplastic right lung with multiple clustered lung cysts of peripheral and peribronchovascular distribution, likely due to recurrent infective processes, and left lung hyperinflation, pulmonary plethora, and focal consolidation of the posterobasal segment of the left lower lobe suggestive of acute infective changes (Figures 4 and 5, Video 7).

Intravenous vancomycin was later commenced following the blood culture results. The patient was extubated successfully within 72 hours of admission and then successfully completed the course of intravenous antibiotics with improvement in clinical and biochemical parameters for infection. Prophylactic oral antibiotics were also started following consultation with our infectious disease colleagues. Aside from this, a referral to our only adult congenital heart disease service in the National Heart Institute (located in a different state) was made, but the patient had found it difficult to attend any scheduled appointments due to logistic and financial reasons. The patient continues to be under our outpatient follow-up with occasional, remote consultation with the adult congenital heart disease services in the National Heart Institute of Malaysia.

## Discussion

Unilateral absence of pulmonary artery is a rare congenital condition first described in 1868 and occurs in 1 in 200,000 adults.^1^ It occurs due to altered development of the sixth segment of the aortic arch with pulmonary artery agenesis.^1^^,^^2^ When affecting the left trunk, it commonly is associated with other congenital defects, whereas RPA involvement often exists in isolation.^2^ About two-thirds of cases involve the RPA.^3^ In a retrospective review of 108 patients with UAPA, clinical presentations varied among individuals, but approximately 40% presented with chest pain, pleural effusions, recurrent infections, dyspnea, reduced effort tolerance, or pulmonary hypertension.^4^ Hemoptysis occurred in 20% of patients, and the overall mortality rate was 7%, with individuals dying from massive pulmonary hemorrhage, respiratory failure, or cor pulmonale.

Initial chest radiography can provide hints including oligemia and volume loss of the ipsilateral lung, with compensatory hyperinflation and plethora on the contralateral side.^1^^,^^3^ Although conventional pulmonary angiography remains the gold standard, CT imaging of the thorax is often sufficient to confirm the diagnosis of structural absence of the affected pulmonary trunk, alongside lung sequelae including bronchiectasis, cystic changes, or air trapping.^5^ Development of bronchiectasis is poorly understood at present, with various mechanisms being proposed, including bronchoconstriction from alveolar hypocapnia and poor mucocilliary clearance following impaired circulation of inflammatory cells predisposing to mucus trapping. Hypoperfusion from absence of the artery may also lead to poor lung parenchymal development with subsequent scarring and cystic changes.^6^

Although the role of echocardiography in UAPA is often limited to detection of features suggestive of pulmonary hypertension on serial monitoring, structural absence of the RPA can occasionally be appreciated using the suprasternal window when technically obtainable, as seen in our patient.^3^^,^^5^ However, such windows are often difficult to obtain in most adults, highlighting the need to consider multimodality imaging when suspicion remains high. An additional benefit to performing TTE in cases of UAPA, specifically in those affecting the left trunk, is being able to detect associated congenital defects.^3^^,^^5^ Unfortunately, in our case, the only available imaging modality was both the TTE and an outsourced CT thorax. Cardiovascular magnetic resonance imaging, which is often useful in describing morphological features of congenital anomalies, could not be performed in our resource-limited setting. However, from the TTE there were no overt features to suggest the presence of other congenital anomalies including commonly associated conditions such as tetralogy of Fallot, atrial and ventricular septal defects, or truncus arteriosus.^7^

Management of an isolated UAPA often depends on the presence or absence of hemoptysis and pulmonary hypertension and can include selective embolization of collateral arteries, pneumonectomy, or use of pulmonary hypertension medications.^6^ Other options include early revascularization in young patients, which can influence the trajectory of the disease and circumvent the development of pulmonary hypertension. However, in older patients, this is seldom encouraged as intrapulmonary arteries are often severely stenosed or obstructed due to fibrotic changes.^6^

## Conclusion

In cases of recurrent bronchopneumonia and bronchiectasis, the presence of an alternative diagnosis such as UAPA, despite being uncommon, should be considered. Our case is unique as UAPA remains an uncommon diagnosis and initial diagnosis through TTE remains underreported. In communities where advanced cardiac imaging is not readily available, chest radiography and echocardiography are pivotal in raising suspicion of the diagnosis of UAPA and provide a guide for prioritizing outsourced investigations like CT imaging.

## Consent Statement

Complete written informed consent was obtained from the patient (or appropriate parent, guardian, or power of attorney) for the publication of this study and accompanying images.

## Ethics Statement

The authors declare that the work described has been carried out in accordance with the following guidelines: Ethics approval was waivered by the Universiti Teknologi MARA (UiTM) Sungai Buloh. Ethics Committee due to the nature of the manuscript (i.e. case report).

## Funding Statement

The authors declare that this report did not receive any specific grant from funding agencies in the public, commercial, or not-for-profit sectors.

## Disclosure Statement

The authors report no conflicts of interest.

## Supplementary Data

Video 1 Two-dimensional TTE, without and with color-flow Doppler, basal parasternal short-axis (PSAX; *top left*), apical 4-chamber (*top right*), mid-PSAX (*bottom left*), and parasternal long-axis (*bottom right*) views, demonstrates normal LV systolic function (LV ejection fraction 56%), normal wall thickness, and normal right heart dimensions. No valve regurgitation or stenosis was seen.

Video 2 Two-dimensional TTE without (*left*) and with (*right*) color-flow Doppler, suprasternal view, demonstrates the LA, descending aorta, and absence of the RPA.

Video 3 Two-dimensional TTE, right ventricular inflow view during diastole and systole, without (*left*) and with (*right*) color-flow Doppler, demonstrates normal right atrial and ventricular dimensions, with no overt valvular lesions and mild tricuspid regurgitation.

Video 4 Two-dimensional TTE, subcostal view during diastole and systole, demonstrates normal atrial and ventricular dimensions.

Video 5 Two-dimensional TTE without (*left*) and with (*right*) color-flow Doppler, suprasternal window (crab view), demonstrates the left pulmonary veins were visible but there was absence of the right pulmonary veins. The RPA was also notably absent.

Video 6 Two-dimensional TTE, superior parasternal short-axis view with color-flow Doppler, demonstrates the missing divergent flow that should be seen from the main pulmonary artery to the RPA.

Video 7 Chest CT with contrast, axial stack displayed from cranial to caudal, revealed filling defects where an RPA should arise from the main pulmonary artery trunk.
